# Acute encephalopathy in children with tuberous sclerosis complex

**DOI:** 10.1186/s13023-020-01646-8

**Published:** 2021-01-06

**Authors:** Shingo Numoto, Hirokazu Kurahashi, Atsushi Sato, Masaya Kubota, Takashi Shiihara, Tohru Okanishi, Ryuta Tanaka, Ichiro Kuki, Tetsuhiro Fukuyama, Mitsuru Kashiwagi, Mitsuru Ikeno, Kazuo Kubota, Manami Akasaka, Masakazu Mimaki, Akihisa Okumura

**Affiliations:** 1 Department of Pediatrics, Aichi Medical University, 1-1 Yazako Karimata, Nagakute, Aichi 480-1195 Japan; 2 Department of Pediatrics, The University of Tokyo Hospital, Tokyo, Japan; 3 Division of Neurology, National Center for Child Health and Development, Tokyo, Japan; 4 Department of Neurology, Gunma Children’s Medical Center, Shibukawa, Gunma Japan; 5 Department of Child Neurology, Seirei Hamamatsu General Hospital, Hamamatsu, Japan; 6 Department of Child Health, Ibaraki Pediatric Education and Training Station, University of Tsukuba, Mito, Japan; 7 Department of Pediatric Neurology, Osaka City General Hospital, Osaka, Japan; 8 Division of Neurology, Nagano Children’s Hospital, Nagano, Japan; 9 Department of Pediatrics, Hirakata City Hospital, Osaka, Japan; 10 Department of Pediatrics, Faculty of Medicine, Juntendo University, Tokyo, Japan; 11 Department of Pediatrics, Gifu University Graduate School of Medicine, Gifu, Japan; 12 Department of Pediatrics, School of Medicine, Iwate Medical University, Morioka, Japan; 13 Department of Pediatrics, Teikyo University School of Medicine, Tokyo, Japan

**Keywords:** Clinical neurology history, Prognosis, Status epilepticus, Infantile spasms, MRI

## Abstract

**Objective:**

We examined the clinical manifestations of acute encephalopathy (AE) and identify risk factors for AE in children with tuberous sclerosis complex (TSC).

**Methods:**

The clinical data of 11 children with clinically diagnosed TSC associated with AE and 109 children with clinically diagnosed TSC alone aged 4 years or older were collected from 13 hospitals.

**Results:**

Of the 11 children with AE, 5 had histories of febrile seizures (FS), and all had histories of febrile status epilepticus (FSE). AE developed within 24 h after fever onset in all children with seizures lasting 30 min or longer. All children developed coma after seizure cessation. Head magnetic resonance imaging (MRI) revealed widespread abnormalities in the cerebral cortex, subcortical white matter, corpus callosum, basal ganglia, and thalamus. One child died; seven had severe neurological sequelae; and the other three, mild sequelae. Logistic regression analysis revealed that a history of FSE was correlated with the development of AE.

**Significance:**

AE in children with TSC was characterized by sudden onset after fever, followed by coma, widespread brain edema evident on MRI, and poor outcomes. A history of FSE was a risk factor for the development of AE.

## Introduction

Tuberous sclerosis complex (TSC) is an autosomal-dominant genetic disorder caused by mutation of the *TSC1* or *TSC2* gene [1], characterized by multiple hamartomas in the skin, brain, heart, kidney, and lungs [2, 3]. A hyperactive mammalian target of rapamycin (mTOR) pathway plays a key role in the pathophysiology of TSC and seizure development in patients with the condition [4, 5]. TSC is one of the major genetic causes of epilepsy; about 85% of TSC patients present with seizures [6–8], especially in infancy [9]. Patients with TSC may exhibit multiple types of seizures refractory to antiepileptic drugs (AEDs) [10, 11]. Many authors have evaluated epilepsy in patients with TSC, but little is known about acute encephalopathy (AE) [12–14], which is characterized by impaired consciousness with or without other neurologic findings such as seizures lasting for > 24 h. We encountered a boy with TSC complicated by AE. He had a history of epileptic spasms followed by focal seizures, but the seizures were well controlled by vigabatrin and carbamazepine. His psychomotor development was slightly delayed, and he exhibited mild autistic features. At 16 months of age, he developed febrile status epilepticus (FSE). The seizure lasted for 35 min, whereas complete recovery of consciousness was seen within 8 h and no neurological sequelae was recognized. At 23 months of age, he developed AE. Although he received intensive care, marked brain edema developed, followed by brain herniation. EEG revealed generalized slowing, followed by extremely low voltage EEG activity. He died 20 days after AE onset. We discussed this child with other pediatric neurologists and found that similar cases had been observed in other hospitals. This prompted us to investigate AE in children with TSC.

This study examined the clinical manifestations of AE to identify risk factors for AE in children with TSC. We presumed that excitotoxicity attributable to prolonged seizures is the principal cause of the irreversible brain lesions. A better understanding of the clinical manifestations of AE will aid clinicians in treating children with TSC, as early identification of the problem will facilitate appropriate treatment. Identification of children at high risk is essential to ensure that caregivers of children with TSC receive appropriate information. We thus performed a retrospective multicenter study.

## Materials and methods

This study was approved by the Ethics Committee of Aichi Medical University Hospital. TSC was clinically diagnosed using the criteria of the 2012 International TSC Consensus Conference [14]. In all patients, two or more measure features were recognized, fulfilling the criteria of the definite diagnosis of TSC, although genetic analysis was rarely performed. We formed a research group to clarify the clinical features of and risk factors for AE in children with TSC. We invited researchers to join the group using the mailing list of the Annual Zao Conference on Pediatric Neurology (http://sites.google.com/site/zaoseminar/). The mailing list includes more than 1,000 pediatric neurologists from all over Japan. First, in November 2017, the senior author (AO) commenced enrolment of patients with TSC who had experienced AE. Here, febrile seizure (FS) was defined as a seizure accompanied by pyrexia of 38 °C or higher without central nervous system infection or acute metabolic derangement lasting for 30 min or less. FSE was defined as a seizure accompanied by pyrexia of 38 °C or higher lasting for 30 min or longer, with recovery of consciousness within 24 h and complete recovery without neurological sequelae. AE was defined as a condition characterized by impaired consciousness with or without other neurologic findings, such as seizures, involuntary movement, and delirious behavior, lasting for > 24 h in children with infection symptoms, including fever, cough, and diarrhea, according to our previous study [15]. Coma was defined as marked loss of consciousness when a patient could not be awakened by painful stimuli, which could not be attributable to sedative effects of the drugs. Eight hospitals, including ours, wherein pediatric neurologists had encountered children with TSC complicated by AE joined the research group. A total of 11 children with clinically diagnosed TSC associated with AE were reported. One of them had been reported elsewhere as a case report [16]. Demographic information, disease manifestations and complications of TSC before the onset of AE, any family history of TSC, epilepsy, or FS, clinical manifestations, laboratory data obtained during AE, treatments, and outcomes of the 11 children were collected using a structured questionnaire. In addition, MRI data on all children were obtained. Outcomes were divided into three categories: death, severe sequelae (no verbal communication and/or bedridden), and mild sequelae (capable of verbal communication and sitting unaided).

We also collected clinical data on children with clinically diagnosed TSC who had not experienced AE to assess the risk factors for AE in children with TSC. As mentioned below, the age at onset of AE was less than 4 years in all but one child, suggesting that children with TSC who are younger than 4 years of age may develop AE in the future, even if they had no history of AE. Therefore, we excluded children with TSC under 4 years of age from the control group. The 106 control children who were 4 years of age or older and who had never had AE were recruited from 13 hospitals: the aforementioned eight hospitals that had seen children with AE and 5 additional hospitals. We retrospectively collected the following information: sex, any history of FS, FSE, and non-febrile SE, any family history of TSC, epilepsy and FS, and complications of TSC such as subependymal giant cell astrocytoma (SEGA), cognitive disorder, any epilepsy, epileptic spasms, and focal seizures observed by 4 years of age. The genetic data were not evaluated because genetic examinations were performed only in a few patients, so we could not discuss the difference between genetic diagnosis and clinical diagnosis.

All statistical analyses were performed using EZR ver. 1.37 (http://www.jichi.ac.jp/saitama-sct/SaitamaHP.files/statmed.html) [17]. A *p*-value < 0.05 was considered to indicate statistical significance. To define risk factors for AE in children with TSC, we first compared clinical variables between children with and without AE using Fisher’s exact test. Then, we performed logistic regression analysis to identify contributors to the occurrence of AE. We used stepwise selection based on *p*-values to identify risk factors for the occurrence of AE.

## Results

### Clinical features of AE in children with TSC

Table 1 lists the demographic features of children with AE. The median age at AE onset was 22 months (range, 16–52 months). All but one child developed AE before 4 years of age. Five children had histories of FS and all had histories of FSE. Ten children had epilepsy and nine had epileptic spasms. Cognitive disorders were evident in six children before AE onset.

**Table 1:** Demographic features of patients with acute encephalopathy

Case	1	2	3	4	5	6	7	8	9	10	11
Age at AE (months)	23	46	16	17	29	22	21	22	34	52	16
Sex	Male	Male	Male	Male	Female	Female	Female	Female	Male	Female	Female
Prior history											
History of FS	Yes	No	No	Yes	Yes	No	No	No	Yes	Yes	No
History of FSE	Yes	No	No	Yes	Yes	No	No	No	Yes	Yes	No
History of non-febrile SE	No	No	No	No	No	No	Yes	No	Yes	No	No
Family history											
Family history of TSC	No	Yes	Yes	No	Yes	No	No	Yes	No	No	No
Family history of FS	No	No	No	No	Yes	No	No	No	No	No	No
Family history of epilepsy	No	Yes	No	No	Yes	No	No	No	No	No	Yes
Complication of TSC											
Epilepsy	Yes	Yes	Yes	Yes	Yes	No	Yes	Yes	Yes	Yes	Yes
Epileptic spasms	Yes	Yes	Yes	Yes	Yes	No	Yes	Yes	Yes	No	Yes
Cardiac rhabdomyoma	No	No	Yes	Yes	No	Yes	No	Yes	Yes	Yes	Yes
Renal AML	No	No	No	No	Yes	No	No	No	Yes	Yes	No
SEGA	No	Yes	No	Yes	No	No	No	No	No	Yes	No
ASD	Yes	Yes	Yes	No	No	No	No	No	Yes	No	No
ADHD	No	No	No	No	No	No	No	No	No	No	No
Cognitive disorder	No	Yes	Yes	No	Yes	Yes	Yes	No	Yes	No	No

*AE* acute encephalopathy, *FS* febrile seizures, *FSE* febrile status epilepticus, *SE* status epilepticus, *TSC* tuberous sclerosis complex, *AML* angiomyolipoma, *SEGA* subependymal giant cell astrocytoma, *ASD* autistm spectrum disorder, *ADHD* attention-deficit hyperactivity disorder

Table 2 lists the clinical manifestations of AE, which developed within 24 h of fever onset in all children accompanied by seizures lasting 30 min or longer. All children entered comas after seizure cessation. Laboratory data obtained on arrival showed mild serum elevations of aspartate aminotransferase (AST), creatine kinase (CK), and lactate dehydrogenase (LD) in some children, but renal function tests were normal. Blood glucose and serum ammonia levels were commonly elevated. Cerebrospinal fluid (CSF) analysis was performed in four patients. In all cases, cell counts were not increased (0–3 cells/µL) and protein levels was not elevated (10–19 mg/dL).

**Table 2:** Clinical manifestations of acute encephalopathy

Case	1	2	3	4	5	6	7	8	9	10	11
Fever to AE (h)	23	3	0	4	24	10	6	8	10	4	3
Duration of the initial seizure (min)	30	70	80	60	60	90	115	105	30	50	80
Pathogen (site of detection)	ND	ND	HHV-6 (blood)	HHV-7 (blood)	ND	ND	ND	HHV-6 (blood)	Influenza (nasal swab)	ND	ND
Laboratory data on presentation											
AST (IU/L)	47	47	62	85	46	48	44	30	38	53	161
ALT (IU/L)	13	28	20	25	16	19	12	28	11	20	33
CK (IU/L)	109	135	184	315	78	148	57	85	70	109	227
LD (IU/L)	463	262	319	435	309	299	409	352	296	377	812
BUN (mg/dL)	13.3	14	11.5	8.2	14.4	17.2	18.9	13.6	12	14.3	16.3
Creatinine (mg/dL)	0.32	0.44	0.39	0.33	0.4	0.3	0.44	0.21	0.45	0.51	0.28
Glucose (mg/dL)	232	303	358	219	248	ND	286	329	ND	ND	ND
Ammonia (μg/dL)	ND	256	343	434	320	147	243	103	152	ND	ND
CSF cell count (/µL)	1	ND	ND	ND	2	ND	0	ND	3	ND	ND
CSF protein (mg/dL)	18	ND	ND	ND	10	ND	19	ND	12	ND	ND
MRI-reduced water diffusion	Cx, SWM, and Th	Cx, SWM, BG, and Th	Cx and SWM	Cx and SWM	Cx and BG	Cx, SWM, Th, and CC	Cx, SWM, and CC	Cx and SWM	Cx, SWM, and BG	Cx and SWM	Cx and SWM
Treatment											
Artificial ventilation	Yes	Yes	Yes	Yes	Yes	Yes	Yes	Yes	Yes	Yes	Yes
Steroid pulse therapy	Yes	No	No	Yes	Yes	Yes	Yes	Yes	No	Yes	Yes
IVIG	No	No	No	Yes	No	Yes	No	No	No	No	Yes
Hypothermia	No	No	No	Yes	No	No	No	No	Yes	Yes	No
Use catecholamine	No	No	No	No	No	No	No	No	No	No	No
Outcome	Death	Severe sequelae	Severe sequelae	Severe sequelae	Severe sequelae	Severe sequelae	Mild sequelae	Severe sequelae	Mild sequelae	Severe sequelae	Mild sequelae

*AE* acute encephalopathy, *ND* not determined, *HHV* human herpesvirus, *AST* Aspartate aminotransferase, *ALT* Alanine aminotransferase, *CK* creatine kinase, *LD* lactate dehydrogenase, *BUN* blood urea nitrogen, *Cx* cortex, *SWM* subcortical white matter, *BG* basal ganglia, *Th* thalamus, *CC* corpus callosum, *IVIG* intravenous immunoglobulin

**Fig. 1:**
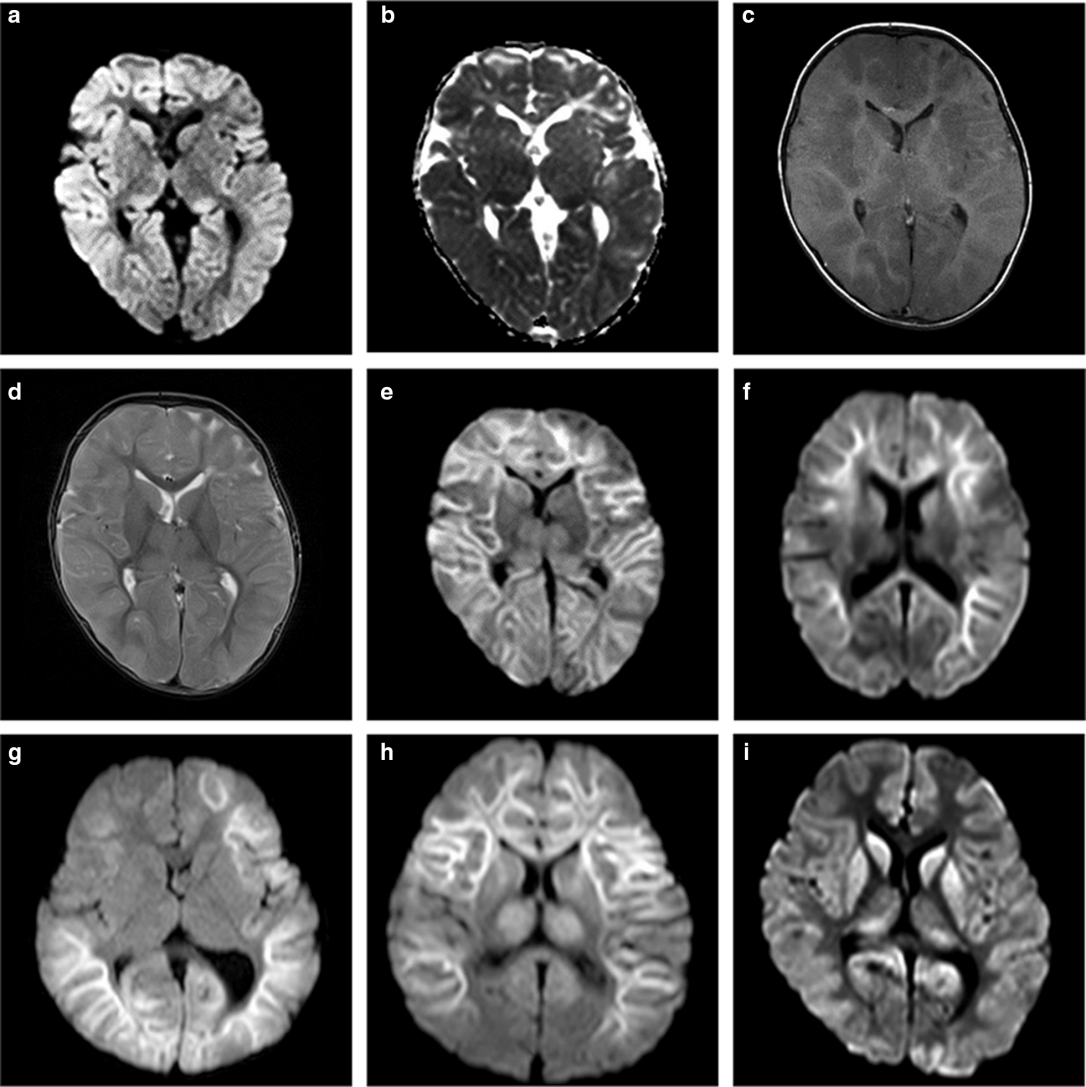
MRI findings. **a**, **b** MRI findings in patient 1 at the onset of acute encephalopathy (AE). Reduced water diffusion was evident over the entire cerebral cortex and the posterior parts of the thalami (**a** diffusion-weighted images; **b** apparent diffusion coefficient map). **c**–**e** MRI of patient 1 performed 4 days after AE onset. Marked brain edema with narrowing of the lateral ventricles was seen on T1- (**c**) and T2-weighted (**d**) images. Diffusion-weighted images exhibited reduced water diffusion, predominantly in the subcortical white matter (**e**). **f** MRI of patient 3 performed 7 days after AE onset. Diffusion-weighted images revealed reduced water diffusion in the subcortical white matter and corpus callosum. **g** MRI of patient 4 performed 3 days after AE onset. Reduced water diffusion was evident in the subcortical white matter of the left frontal and bilateral temporo–parieto–occipital areas. **h** MRI of patient 6 performed 5 days after AE onset. Reduced water diffusion was evident in the bilateral thalami and subcortical white matter, except for the bilateral occipital areas. **i** MRI of patient 2 performed 17 days after AE onset. Diffusion-weighted images revealed reduced water diffusion in the caudate nuclei, basal ganglia, and the pulvinar thalami

Head MRI was performed in all children at 1–17 days after AE onset (Fig. 1). Diffusion-weighted images revealed brain edema, predominantly in the cerebral cortex, associated with reduced water diffusion in the cortex and/or subcortical white matter. MRI performed on the day of onset in one child revealed reduced water diffusion over the entire subcortical white matter. No reduction in water diffusion was seen in the area of the cortical tubers. The corpus callosum, basal ganglia, and thalamus were involved in two, three, and three children, respectively. No lesion was seen in the cerebellum.

All children exhibited monophasic clinical courses, with no seizures after cessation of the initial prolonged seizure. All children required admission to the intensive care unit and artificial ventilation; none required an inotropic agent or treatment of disseminated intravascular coagulation. Steroid pulse therapy was prescribed for eight children, intravenous immunoglobulin for three, and hypothermia for three. One child died, seven had severe neurological sequelae, and the other three showed mild sequelae.

### Risk factors for AE in children with TSC

Table 3 shows the results of univariate analysis. Histories of FS and FSE were more frequent in children with than in those without AE. Family history of FS or epilepsy and TSC disease manifestations did not differ between the two groups. Epileptic spasms were more frequent in children with than without AE, although the rate of epilepsy did not differ between the two groups. SEGA and cognitive disorder did not differ between the two groups.

**Table 3:** Univariate analysis

	Patients with AE (N = 11)	Patients with no AE (N = 106)	*p*-value
Sex (male: female)	5:6	63:43	0.52
History of FS	5 (45%)	17 (16%)	0.032
History of FSE	5 (45%)	14 (13%)	0.017
History of non-febrile SE	2 (18%)	15 (14%)	0.66
Family history of TSC	4 (36%)	23 (22%)	0.28
Family history of FS	1 (9%)	3 (3%)	0.34
Family history of epilepsy	3 (27%)	10 (9%)	0.11
SEGA	3 (27%)	25 (24%)	0.72
Cognitive disorder	6 (55%)	51/103 (50%)	> 0.99
Any epilepsy	10 (91%)	93 (88%)	> 0.99
Epileptic spasms	9 (82%)	48 (45%)	0.027
Focal seizures	8 (73%)	85 (83%)	0.69

*AE* acute encephalopathy, *FS* febrile seizures, *FSE* febrile status epilepticus, *SE* status epilepticus, *SEGA* subependymal giant cell astrocytoma

Table 4 shows the results of multivariate analysis. Three variables (history of FS, history of FSE, and epileptic spasms) had *p*-values < 0.1 on univariate analysis. Multivariate analysis started using these three variables. Logistic regression with stepwise selection excluded a history of FS. Then, logistic regression analysis revealed that a history of FSE was correlated with the development of AE, whereas the association with previous epileptic spasms was not significant after adjusting for the effects of FSE.

**Table 4:** Multivariate analysis

	Odds ratio (95% confidence interval)	*p*-value
History of FSE	4.70 (1.25–17.7)	0.022
Epileptic spasms	2.56 (0.62–10.5)	0.19

*FSE* febrile status epilepticus

## Discussion

We successfully documented the clinical manifestations of AE in children with TSC. AE developed within 24 h after fever onset, followed by a prolonged coma. MRI revealed brain edema with reduced water diffusion, predominantly in the subcortical white matter. Outcomes were poor (severe neurological sequelae or death in most patients) despite various treatments. We also found that a history of FSE was associated with the development of AE.

It is remarkable that the clinical manifestations of AE were similar among the children. All experienced AE within 1 day of fever onset, seizures lasting for 30 min, a monophasic clinical course with coma, and widespread MRI abnormalities. We presume that excitotoxicity attributable to prolonged seizures is the principal cause of the irreversible brain lesions because all children had a prolonged seizure refractory to antiepileptic drugs. Hypercytokinemia may be also involved in AE pathogenesis, but the laboratory abnormalities of our patients were milder than those of patients with acute necrotizing encephalopathy [18, 19], which is considered to be caused by a “cytokine storm” associated with multiorgan failure and disseminated intravascular coagulation. Marked elevations of enzymes such as AST and LD were common in children with acute necrotizing encephalopathy immediately after disease onset [20]. Hypercytokinemia may play only a limited role in the development of AE in children with TSC. On the other hand, elevated blood glucose and serum ammonia levels were common in our patients, suggesting metabolic derangement; this may be a sequela of critical illness caused by AE.

MRI revealed widespread abnormalities in all children. Reduced water diffusion (indicating cytotoxic edema) was evident, predominantly in the subcortical white matter, and conventional MRI suggested edema in the cerebral cortex. A similar MRI pattern is seen in children with Dravet syndrome complicated by AE [15, 21]. Okumura et al. reported 15 such children and showed that brain edema and reduced water diffusion in the cortical and/or subcortical white matter were characteristic of the condition [15]. Notably, a prolonged seizure is an initial symptom of AE in children with Dravet syndrome. Widespread MRI abnormalities with cytotoxic edema may be neuroimaging features of AE in children with TSC.

Initial laboratory abnormalities and CSF analysis abnormalities were mild (or absent) in our children. This implies that the brain disorders of children with AE exhibited sudden onset and rapid progression. All children presented with a seizure induced by fever; distinguishing AE from less severe seizures is clinically difficult on initial presentation. Laboratory data may not be helpful; no marked abnormalities are present. However, hyperglycemia was common in AE children on presentation. Hyperglycemia is correlated with adverse outcomes of status epilepticus and AE [22–24] and may be a convenient predictor of AE.

The outcomes of children with TSC complicated by AE were poor, although intensive treatment was performed. Treatments included supportive efforts to stabilize the general condition, seizure control, and neuroprotection. Although all patients required intensive care and artificial ventilation, their general condition was appropriately maintained. No patient developed shock, serious multiorgan failure, or disseminated intravascular coagulation. Seizure control was achieved in all patients after the aggressive use of antiepileptic drugs. A recent consensus treatment for status epilepticus refers to prompt recognition and the need for very early treatment to reduce morbidity and mortality, drug requirements, and seizure duration [25, 26]. Studies employing buccal or intranasal midazolam found that delivery via non-intravenous routes was a practical, rapid, reasonably safe, and effective alternative to intravenous lorazepam or diazepam as a first-line treatment for early status epilepticus in out-of-hospital settings [27, 28]. No such rescue drugs (example: buccal midazolam) are yet available in Japan. Neuroprotective treatment will be a subject of a future study. Several pharmacological and non-pharmacological treatments including intravenous immunoglobulin, corticosteroids, neuroactive steroids, and hypothermia have been used to treat patients who presented with status epilepticus [29–32], but neither efficacy nor tolerability has been investigated.

Our index case died of AE. Shepherd et al. explored the causes of death of TSC patients and found that 9 of 40 TSC patients who died had status epilepticus [11]. The age at death ranged from infancy to adulthood. Shehata et al. reported that 2 of 21 patients with TSC complicated by status epilepticus died [12]. These reports did not give detailed clinical and genetic information, and it is uncertain whether the dead patients met our criteria for AE. Welin et al., who used national registry data to estimate the prevalence of epilepsy and mortality associated with TSC in Sweden [33]. The causes of death were directly related to TSC in 15 of 30 patients who died, including 3 who died of epilepsy. No additional information was provided. Amin et al. reported that renal disease was a major cause of mortality in TSC patients and for sudden unexpected death from epilepsy [34]. No information on status epilepticus was given. Although the frequencies of AE may be low, more attention should be paid to AE to improve the long-term outcomes of patients with TSC.

We found that a history of FSE was a risk factor for AE in children with TSC. Nearly half of children with AE had experienced FSE before AE onset. Little attention has been paid to the relationship between TSC and FS. No study has adequately investigated the rate or clinical manifestations of FS in children with TSC. Notably, a history of FS in our study was more frequent (16%) in children with TSC but without AE than in the general population (3–8% in Japan). This suggests several different scenarios. One possible explanation is that children with TSC may be intrinsically susceptible to FS. However, no data support this hypothesis. Experimental and/or epidemiological studies are required. Another possibility is that mTOR pathway plays a role in FSE. The association between mutations in mTOR pathway genes and epileptic network has reported, and studies in rodent models of status epilepticus demonstrate that mTOR signaling is activated by status epilepticus [35]. However, this biological hypothesis is unclear because there have been no studies on the relation between mTOR pathway and fever. Another possibility is that genes other than *TSC1*/*TSC2* may contribute to AE development. Mutations in *SCN1A* and *PCDH19* are well known to cause several types of epilepsy that are associated with FS [36, 37]. Mutations in *SCN1B*, *SCN2A*, *SCN9A*, *GABRG2*, *CACNA1H*, and *STX1B* have been found in families exhibiting genetic epilepsy with FS [38]. It is possible that some genetic variants may modify the phenotypes of TSC, increasing susceptibility to FS. It is also possible that initial FSE may precipitate FSE recurrence, increasing the risk of AE in children with TSC. Maytal et al. reported that development of FSE in an otherwise normal child did not increase the risk of subsequent FS during the first few years following the initial episode [39]. By contrast, the FEBSTAT study revealed that the risk of subsequent FSE was significantly increased in those with an initial FSE compared to a simple FS and that any MRI abnormality increased the risk 3.4 fold [40]. These results may support the hypothesis that FSE occurrence may increase the risk of later FSE /AE in children with TSC.

Our study has several limitations. The selection of control children with TSC may have affected the results. A distinct feature of TSC is that disorders of various organs appear at different ages. The clinical manifestations of TSC develop with age, and the extent of each symptom or complication changes constantly. The severity of clinical manifestations varies widely, even in a single patient, according to age. A neonate with TSC may have no epileptic seizures but may have seizures in the future. Similarly, a young infant with no history of AE may develop AE in the future. Therefore, we believe that the clinical variables should be compared at specific ages. We found that the age at the onset of AE in most cases was 4 years of age or younger. Thus, we excluded children with TSC under 4 years of age from the control group and compared clinical variables that were recognized by 4 years of age; the appropriateness of such exclusion may be controversial. The time at which clinical information was collected may affect our results. We could not perform genetic analysis of all children. It is possible that the risk of AE may be correlated with the type of *TSC1*/*TSC2* mutation. Genetic analysis would yield useful information on AE development in children with TSC. Finally, this was a retrospective study with a small number of patients. The results of this study should be validated by prospective studies with more sophisticated designs.

## Conclusion

We clarified the clinical manifestations of AE and risk factors for the condition in children with TSC. AE in such children was characterized by sudden onset after fever and was followed by coma, widespread brain edema evident on MRI, and poor outcomes. A history of FSE was a risk factor for AE development. Our results will be useful when imparting information to caregivers and will aid clinicians who encounter children with TS with a history of FSE.

## Data Availability

Anonymized data and materials can be made available upon reasonable request to the corresponding author.
